# Indoor Air Purification and Residents’ Self-Rated Health: Evidence from the China Health and Nutrition Survey

**DOI:** 10.3390/ijerph19106316

**Published:** 2022-05-23

**Authors:** Lei Li, Yilin Zheng, Shaojun Ma

**Affiliations:** College of Management and Economics, Tianjin University, Tianjin 300072, China; lilei@tju.edu.cn (L.L.); mashaojun0212@tju.edu.cn (S.M.)

**Keywords:** indoor air purification, self-rated health, indoor pollution, China Health and Nutrition Survey

## Abstract

Indoor air pollution is injurious to human health, even worse than outdoor air pollution. However, there is a lack of empirical evidence using large samples in developing countries regarding whether indoor air purification can improve human health by reducing indoor air pollutants. Using the data from the China Health and Nutrition Survey in 2015, this study analyzes the relationship between indoor air purification and residents’ self-rated health. We apply the generalized ordered logit model and find that indoor air purification has a significantly positive effect on residents’ self-rated health. This positive effect is limited to improving the probability of residents’ health level being rated “good”, and there is no significant movement between the two levels of “bad” and “fair”. The results also show that, as an important source of indoor air pollutants, solid fuels used in cooking significantly reduced residents’ self-rated health level. Additional results show the heterogeneity of the relationship between indoor air purification and resident health among groups with different characteristics. This study provides empirical evidence for further optimizing the indoor air environment.

## 1. Introduction

Air pollution that must be settled for the sustainable development of a country, especially in developing countries [1,2,3,4,5]. The global air quality data platform IQAir released the “2020 World Air Quality Report”, pointing out that the global air quality situation is still severe. Among the 106 monitored countries, only 24 countries had reached the annual PM2.5 guideline limits set by the World Health Organization in 2020. Notably, during the COVID-19 epidemic, being exposed to particulate matter pollution makes people more vulnerable to the virus, thus increasing harm to human health [6]. Studies have shown that indoor air pollution is more serious than outdoor air pollution [7]. The concentration of pollutants indoors is five times that of outdoors, and in some cases, it can reach 100 times [8,9]. For example, Vasile et al. [10] showed that the concentration of CO_2_ in kitchens and bedrooms in dwellings can be three times higher than outdoors in Central and Eastern Europe.

Air quality in the indoor environment is an important factor affecting human health [11,12]. People spend an average of 70–80% of their time indoors, and the negative health impacts associated with exposure to pollutants are responsible for high morbidity and mortality worldwide [12,13]. Sources of indoor air pollution include synthetic materials and chemicals in buildings for indoor architectural and decorative purposes, sealants, cleaners, and gases from cooking activities, among others [11,14]. These changes lead to higher concentrations of volatile organic compounds, such as xylene and formaldehyde in the indoor environment [15,16]. Residents’ continued exposure to indoor air pollutants may even lead to respiratory disease, dermatitis, damage to the central nervous system, and certain cancers [17,18,19].

There are various ways to reduce indoor air pollution. These ways include: removing pollution at source [20]; reducing indoor pollutants using air-cleaning technologies, such as biological filtration and plant systems for indoor air purification [1,21,22], and using air purifiers to absorb and filter organic pollutants [23]; improving living conditions by optimizing ventilation and air conditioning systems [24,25]; and reducing smoke exposure by improving kitchen design and changing cooking methods [26]. While reducing sources of contamination is generally the preferred method, it is often difficult to achieve. Furthermore, methods such as ventilation are constrained by outdoor air quality, and it is becoming increasingly difficult to have clean outdoor air in many parts of the world [27]. Although indoor plant systems are beneficial to human health [28], the use of plants to metabolize pollutants is less valuable for indoor air purification due to low absorption rates [29]. Furthermore, the efficiency of indoor plants in removing pollutants is affected by environmental factors [30,31,32].

Air purifiers can significantly reduce indoor pollution concentrations through filtration, adsorption, sterilization, oxidation, etc. This is one of the most commonly used effective methods and has received increasing attention [27,33,34]. However, existing studies on the health effects of indoor air purifier use are mostly randomized crossover trials with few participants [35]. Furthermore, existing research remains limited in the context of indoor air quality in developing countries [11]. The present study focuses on the role of air purifiers, using a wider range of data from the China Health and Nutrition Survey, focusing on the effect of indoor air purification on residents’ self-rated health, aiming to provide new insights and empirical evidence for improving the indoor air environment and residents’ health.

## 2. Literature Review

### 2.1. Air Purifiers and Health

Air purifiers are widely used to reduce indoor air pollution [23]. Most researchers believe that air purifiers are effective in reducing indoor PM2.5 and improving population health [36,37,38,39]. Dong et al. [40] found substantial respiratory benefits from ionized air purifiers. The active intervention of air purifiers in reducing household air pollutants helps to improve allergic airway disease [41]. Air purifiers improve circulatory and cardiorespiratory fitness in older adults [15,42]. Wang et al. [34] found that indoor high-efficiency particulate air filters (HEPA) also have protective effects on cardiorespiratory health among young healthy adults, as reflected in lower blood pressure and systemic oxidative stress, as well as improved lung function. Some scholars have studied the air quality of medical institutions during the COVID-19 pandemic and found that air purifiers have a positive impact on indoor air quality, which can reduce the levels of PM2.5 and total volatile organic compounds (TVOC) [43], thus helping to control airborne SARS-CoV-2 [37].

However, some scholars believe that the effect of air purifiers on health is not clear [44]. Due to the various limitations of existing technologies, the current situation regarding indoor air purification is not ideal [45]. Yoda et al. [46] conducted a randomized crossover intervention study on 32 individuals and found that air purifiers slightly controlled indoor PM2.5 concentrations in households, but had no significant effect on improving health. Alshawa et al. [47] found that some ionized air purifiers may increase fine and ultrafine concentration in an unsaturated VOCs environment. Niu et al. [48] found that indoor air filtration may lead to increased levels of airborne endotoxins.

Mannan and Al-Ghamdi [11], by reviewing scientific studies of the indoor air quality for residential buildings in different regions of the world, found that direct comparisons of indoor air pollutant levels is difficult because assessments are made at different time periods, using different equipment and techniques, and in different types of indoor environments. However, the present study uses data from the China Health and Nutrition Survey with a larger geographic scope to examine the impact of air purifier use on residents’ health and to provide evidence for a comparative analysis of different heterogeneous groups.

### 2.2. Air Pollution from Cooking Activities and Health

The indoor environment is mainly polluted by the interior decoration and the combustion process(es) used in the kitchen [49], with the air pollution from cooking activities being particularly serious. PM10 levels below 2000 μg/m^3^ over an average 24-h period are common, but the peak PM10 exposures have been reported to be over 30,000 μg/m^3^ during cooking [50]. These kinds of emissions are a major cause of respiratory infections, impaired lung growth, lung cancer, and cataracts [51,52,53]. The results of a survey of indoor air in local residential units in Hong Kong showed that the concentration of CO_2_ and PM10 in cookhouse was 14% and 67% higher, respectively, than in living rooms [54]. A survey as part of a regular review in England and Wales found that gas cooking systems were the main factors contributing to high levels of VOC gas emissions [55]. In a housing gas and particulate matter assessment study from the UAE, attached garages, kitchens, and central air-conditioning systems were found to be the main causes of indoor PM2.5 and PM10 [56]. Households burning biomass and charcoal have been found to have higher levels of PM10 and CO compared with households using cleaner fuels [57]. The unvented burning of solid fuels for home cooking and heating has serious health impacts and increased mortality in rural households in developing countries [58].

Nearly three million people worldwide burn fuels (wood, charcoal, and animal dung) for cooking, lighting, and heating, using inefficient stoves in poorly ventilated homes [59]. Solid fuel use is not only occurring in developing countries; this happens in families of high-income countries, too [27]. Therefore, while analyzing the relationship between indoor air purification and residents’ health, the present study also considers the pollutants produced by cooking activities among indoor air pollutants, especially those caused by solid fuel combustion.

## 3. Materials and Methods

### 3.1. Data

The data used in this study are from the China Health and Nutrition Survey (CHNS) data, provided by the Nutrition and Health of the Chinese Center for Disease Control and Prevention. CHNS data were obtained using a multistage, random cluster sampling method to survey a wide range of information on individuals, households, and communities in 15 provinces in China over a period from 1989 to 2015. Furthermore, the research team has not released data of the survey for the latest year. This multistage, random cluster sampling process is a multistage sampling based on administrative divisions. Counties in 15 provinces were stratified by income (low, medium, high), and four counties in each province were randomly selected using a weighted sampling method. Furthermore, these four counties cannot belong to the same city. In addition, the sampling method is a random cluster sampling, which means that the villages and towns in the county and the urban/suburban streets in the city are randomly selected. The investigation team began collecting data on household ownership of air purifiers in 2015; therefore, we only used cross-sectional data from 2015. Observations with missing values in any variable were removed. The final sample included 9612 observations and 4780 households.

### 3.2. Variables

#### 3.2.1. Dependent Variable

In this study, the dependent variable is the self-rated health status. The “Disease History” section of the questionnaire asked the question “Right now, how would you describe your health compared to that of other people your age?”, and respondents made a choice among the following six options: “1. Very good”; “2. Good”; “3. Fair”; “4. Bad”; “5. Very bad”; and “9. Unknown”. We refer to Sun et al. [60] to combine “Very bad” and “Bad” into one group, and then classify them as “Bad” and assign a value of 1. We also combine “Very good” and “Good” into one group and classify them as “Good”, assigning a value of 3. We assign the option “Fair” a value of 2. The selection of “Unknown” was excluded from the analysis. Although the residents’ self-rated health is subjective, it is considered a valid comprehensive assessment of a person’s state of health because it is closely related to a large variety of objective health-level indicators in China [61]. Therefore, the self-rated health status is widely used to measure the health level of individuals [60,62,63].

#### 3.2.2. Explanatory Variables

The core explanatory variable of this study is the number of household air purifiers. In the household questionnaire, statistics were obtained for household appliances, including the number of air purifiers. We also considered possible sources of endocrine-disrupting compounds due to indoor living conditions. Combined with analysis of the literature review and the availability of questionnaire data, we analyzed the health effects of some interesting indoor living condition variables, including the housing size, the type of fuel used, and the type of lighting [63].

#### 3.2.3. Control Variables

In order to study the health effects of indoor air purifiers, we controlled for a range of individual, household, and community-level characteristics, including demographic characteristics (age, gender, marital status, and household size), socioeconomic status (educational level, work status, and per capita household income level), individual health beliefs (smoking, drinking, participation in physical activities, utilization of preventive health care, and participation in medical insurance), and community enablers (city living, urbanization index, and sanitation index) [61,64,65,66,67,68,69]. The urbanization index is based on the diverse characteristics of communities. (for details, see [70]). The sanitation index is mainly calculated from the dimensions of the surrounding sanitary conditions and the proportion of households with treated water. The detailed coding rules and descriptive statistics are shown in Table 1, which shows that about 3.844% of the respondents have air purifiers in their houses. The overall average score of the self-rated health is 2.505, indicating that Chinese residents’ evaluation of their own health was between “Fair” and “Good”.

### 3.3. Model

This study uses the logit model to analyze the health effects of indoor air purification. Since the residents’ self-rated health status is an ordinal variable, we use an ordinal logit model [71,72,73]. The established benchmark model is as follows:

(1) $$Health\_self_{i}^{*}=\beta {\left(Air\_purifier\right)}_{i}+\gamma_{1}{\left(HHarea\right)}_{i}+\gamma_{2}{\left(Fuel\right)}_{i}\;+\gamma_{3}{\left(Lighting\right)}_{i}+\theta X_{i}+\varepsilon_{i}\;$$

where $Health\_self_{i}^{*}$ represents the self-rated health level of resident i, air_purifier represents the number of air purifiers in the household, HHarea represents the housing size (area) of the household, Fuel represents the type of fuel used in the house, Lighting represents the type of lighting used in the house, X is a set of control variables that represent other characteristics of residents and their families and communities, and ε is a random error term.

In reality, we could not obtain specific value of residents’ self-rated health level ($Health\_self^{*}$). Thus, we only observe the rating of residents’ self-rated health level (Health_self): let Health_self = 1, 2, and 3 represent “Bad”, “Fair”, and “Good”, respectively. Residents choose the most suitable option according to their own conditions. This process can be described as:

(2) $$\begin{array}{c} Health\_self=1\;(\mathrm{Bad}),\;\mathrm{if}\;Health\_self^{*}\leq \mu_{1};\\ Health\_self=2\;(\mathrm{Fair}),\;\mathrm{if}\;\mu_{1}<Health\_self^{*}\leq \mu_{2};\;\\ Health\_self=3\;(\mathrm{Good}),\;\mathrm{if}\;Health\_self^{*}>\mu_{2}. \end{array}$$

where $\mu_{1}<\mu_{2}$ represents cutpoints, which are parameters to be estimated together with β, γ, and θ. Assuming that ε obeys the standard logistic distribution, the regression equation can be written in the following form:

(3) $$\begin{array}{cc} \log[\mathrm{Pr}(Health\_ & self_{i}>j|x)/\mathrm{Pr}(Health\_self_{i}>j|x)]\\ & =-\mu_{j}+\beta {\left(Air\_purifier\right)}_{i}+\gamma_{1}{\left(HHarea\right)}_{i}+\gamma_{2}{\left(Fuel\right)}_{i}\\ & +\gamma_{3}{\left(Lighting\right)}_{i}+\theta X_{i},\;j=1,\;2. \end{array}$$

β is the parameter of interest to us, representing the effect of changes in the number of air purifiers on residents’ health. When the number of air purifiers is increased by 1 unit, the odds ratio of increasing the health level of residents by one level or more becomes the original exp (β) times. $\gamma_{1}$, $\gamma_{2}$, $\gamma_{3}$, and θ represent the influence on residents’ health of the housing size, the type of fuel used, the type of lighting, and control variables, respectively.

However, an implicit assumption of the ordinal logit model is that each unit increase in the independent variable has the same effect on the change in the rank of the dependent variable, except for the constant term. Therefore, we tested the parallel regression hypothesis using the Brant test and the likelihood ratio test. The results of the Brant test showed that the assumption of parallelism of the overall equation was rejected at the 1% level (χ^2^ = 61.39, *p*-value = 0.000). The likelihood ratio test also showed the same result (χ^2^ = 61.39, *p*-value = 0.000), which means that independent variables have different degrees of influence on the self-rated health status at different levels. Therefore, we considered using the generalized ordered logit model [60,62,74], which can analyze the influence of independent variables on the dependent variable as a function of the threshold. The established model is as follows:

(4) $$\begin{array}{cc} \log[\mathrm{Pr}(Health\_ & self_{i}>j|x)/\mathrm{Pr}(Health\_self_{i}>j|x)]\\ & =-\mu_{j}+\beta_{j}{\left(Air\_purifier\right)}_{i}+\gamma_{1j}{\left(HHarea\right)}_{i}+\gamma_{2j}{\left(Fuel\right)}_{i}\\ & +\gamma_{3j}{\left(Lighting\right)}_{i}+\theta_{j}X_{i},\;j=1,\;2, \end{array}$$

In Equation (4), when the number of air purifiers increases by 1 unit, the odds ratio of the residents’ health level to be improved to “Fair” becomes exp ($\beta_{1}$) times that of the original level, and the odds ratio to be improved to “Good” becomes exp ($\beta_{2}$) times that of the original level.

In order to ensure the reliability of the regression results, this study also uses the ordered probit model and the generalized ordered probit model for robustness analysis. The logit model assumes that ε obeys standard logistic distribution, while the probit model assumes that ε obeys the standard normal distribution. Therefore, the relationship between the dependent variable and its latent variable is shown as follows:

(5) $$Health\_self_{i}=F\left(Health\_self_{i}^{*}\right)=\{\begin{array}{c} 1,\;Health\_self_{i}^{*}\leq \mu_{1}\\ 2,\;\mu_{1}<Health\_self_{i}^{*}\leq \mu_{2}\\ 3,\;Health\_self_{i}^{*}>\mu_{2} \end{array}$$

where F(·) is the cumulative probability analysis function of the standard normal function. By solving the marginal efficiency effect, the parameters to be estimated in the ordered probit model can be obtained.

(6) $$\frac{\partial (\mathrm{Pr}(Health_{self}{}_{i}=j|x_{k})}{\partial x_{k}}=\left[f\left(\mu_{j}-X^{\prime }\delta \right)-f\left(\mu_{j+1}-X^{\prime }\delta \right)\right]\delta_{k}$$

where $X^{\prime }$ represents the independent variable vector, and $X=\left(air\_purifier,HHarea,\;Fuel,\;Lighting,X_{i}\right)$. δ represents the coefficient vector of the independent variable. The generalized ordered probit model can analyze the influence of independent variables on dependent variables with the change in threshold. The calculated marginal efficiency effect is shown as follows:

(7) $$\frac{\partial (\mathrm{Pr}(Health_{self}{}_{i}=j|x_{k})}{\partial x_{k}}=f\left({\tilde{\mu }}_{j}-X^{\prime }\delta_{j}\right)\times \delta_{j,k}-f\left({\tilde{\mu }}_{j+1}-X^{\prime }\delta_{j}\right)\times \delta_{j,k}$$

## 4. Results and Discussion

In this section, we report the results of the logit model. In Section 4.1, we use the generalized logit model and the generalized ordered logit model to study the impact of indoor air purification on residents’ health. In Section 4.2, we discuss the heterogeneity among residents with different characteristics using group comparisons. Finally, we discuss the results of robustness checks in Section 4.3.

### 4.1. The Effect of Indoor Air Purification and Self-Rated Health

Table 2 reports the results of regression using an ordinal logit model. Column (1) of Table 2 shows the regression results without adding control variables. Columns (2), (3), and (4) represent the regression results of gradually adding control variables, including indoor endocrine-disrupting compounds, personal and family characteristics, and community enablers, respectively. In the regression results of the above four models, the coefficient of indoor air purification is positive and significant, indicating that indoor air purification has a positive impact on self-rated health. However, with the addition of other control variables, the significance level of the coefficient of indoor air purification gradually decreases, indicating that the heterogeneity of individuals affects their judgment of the impact of indoor air purification on self-rated health. It can be seen from the columns (1) and (4) of Table 2 that, if the number of indoor air purifiers increases by one unit, the self-rated health level increases to 1.660 times [=exp(0.507)] and 1.247 times [=exp(0.221)] the original level.

Among the related variables for indoor endocrine-disrupting compounds, the coefficient of housing size is significantly positive, indicating that the larger the housing size, the higher the self-rated health level. The possible reasons are that, on the one hand, the larger the indoor space of the house, the lower the density of endocrine-disrupting compounds, which has a positive impact on the self-rated health level; on the other hand, housing is a high-expenditure item in household consumption, and a large housing size also suggests relatively high family wealth, resulting in a relatively high self-rated health level, which is consistent with the regression results of per capita income. The coefficient of solid fuel is significantly negative, indicating that indoor use of solid fuel causes more serious indoor pollution and can reduce residents’ self-rated health level. The direction of influence of these variables is consistent with previous expectations.

According to the results in Table 2, among the control variables in the demographics category, the coefficient of age is significantly negative, indicating that the self-rated health status of the elderly is relatively poor. The coefficient of married is significantly positive, indicating that married residents are happier, and their self-rated health level is better. However, with the gradual increase in the family population, the family burden becomes heavier, and the residents’ self-rated health status worsens. This conclusion comes from the coefficient result of family size. Among the control variables in the socioeconomic status category, people with higher education, higher income, and jobs have higher self-rated health levels. These results are consistent with our expectations. Regarding the control variables classified according to individual health beliefs, the higher the participation in physical activities, the better the residents’ physical function; thus, they have a better self-rated health level. The coefficients of the utilization rate of preventive health care and participation in medical insurance are significantly negative, indicating that residents with poor self-rated health may choose to join preventive health care and medical insurance. Among the control variables in the category of community-enabling factors, the coefficient of sanitation score was significantly positive, indicating that a higher community sanitation score has a positive impact on individuals’ self-rated health.

**Figure 1:**
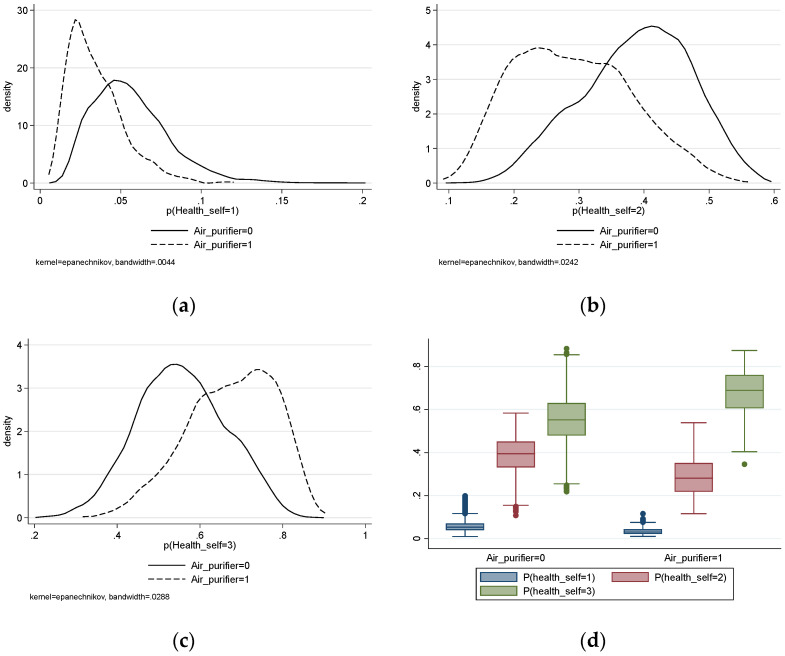
The plots for the predicted probability of self-rated health level: (**a**) kernel density plot of predicted probability for health_self = 1; (**b**) kernel density plot of predicted probability for health_self = 2; (**c**) kernel density plot of predicted probability for health_self = 3; (**d**) boxplot of predicted probabilities for health_self.

However, two methods (the Brant test and likelihood ratio test) were used to test the parallel regression assumption of the ordered logit model, and the results were rejected, indicating that the regression results of the ordered logit model are biased. In addition, we drew the kernel density plot and the boxplot for the predicted probability of self-rated health level (Figure 1). Figure 1a–c shows that the variation trend and the peak value of self-health prediction probability are significantly different with different values of air purification. It can be seen from Figure 1d that the center position and spread range of self-health prediction probabilities of different groups are also significantly different. Therefore, we examine the effect of indoor air purification using the generalized ordered logit model as an analytical tool. The estimated results are shown in Table 3.

The coefficients in the (i) columns in Table 3 represent the results of comparing the self-rated health level between the two groups of “Bad” and “Fair or Good”. The coefficients in the (ii) columns represent the results of comparing the estimated self-rated health level between the “Bad or Fair” and “Good” groups. Table 3 shows that the coefficients are different for each group, so the generalized ordered logit model cannot be simplified into a traditional model, and the estimated results of the generalized ordered logit model are more effective. In the above four models, the coefficients of indoor air purification are all positive, but the effect is different. According to the results of column (4), an increase in the number of air purifiers by one unit will change the odds ratio of the residents’ self-rated level to “Fair”, which is 1.364[=exp (0.311)] times that of the original level times, and to “Good”, which is 1.236[=exp (0.212)] times that of the original level. After adding control variables, the coefficient of indoor air purification on the self-rated health level “Good” is significant at the 10% level, while the coefficient on “Fair” is not significant. This result shows that the promotion effect of indoor air purification on self-rated health levels has little impact on movements between “Bad” and “Fair”, and is mainly reflected in the improvement to the “Good” level. The same grouping differences are also reflected in the coefficients of solid fuels. According to the results in column (4), the coefficient of the effect of solid fuel use on the self-rated health level “Good” is significant at the 5% level, while the coefficient on “Fair” is not significant. This result indicates that the use of solid fuels negatively affects the self-rated health level, mainly by reducing the “Good” level. Both sets of coefficients for housing size were significantly positive, suggesting that a larger housing size has a positive effect on self-rated health. Moreover, the coefficient of the effect of housing size on the self-rated health level “Good” is smaller than the coefficient on “Fair”, indicating that an increase in housing size has a greater impact on the residents’ health level being improved to “Fair” than to “Good”.

The signs and significance of the control variable coefficients in Table 3 are basically consistent with the results in Table 2, but there are still differences in the absolute values of the coefficients in different groups. The variables that have a greater impact on the health level of residents in the “Good” group than in the “Fair” group include married, family size, Edhigh, activities, and medical insurance (most of which are related to family characteristics). The variables that have a greater effect on the individual health level of the “Fair” group than the “Good” group include age, work, smoke, preventive health care, and income (most of which are related to the characteristic behavior of individuals).

### 4.2. Heterogeneity Analysis

The research in the previous section analyzed the effect of indoor air purification on residents’ self-rated health. According to the results in Table 2 and Table 3, we find that after adding control variables, although the effect of indoor air purification on residents’ self-rated health level is still significant, the degree (absolute value of the coefficient) and significance of the effect are decreasing. Zhao et al. [75] showed that individuals from different sociodemographic groups reacted differently to health information feedback. Therefore, this study conducted further group comparisons based on individual characteristics to examine the heterogeneous effects of indoor air purification on the self-rated health of these different groups.

The grouping factors not only include the objective environment of the local community, but also subjective characteristics, such as personal socioeconomic status and health beliefs. We compare the differences between rural and urban residents from an objective perspective. There are obvious differences in the construction materials and interior decoration of rural housing and urban housing, especially the fuel used [58], which leads to differences in their perception of the effect of air purifiers. From a subjective perspective, we selected factors, including education level, working, and smoking for heterogeneity analysis [60]. Consumers’ risk perception and product understanding knowledge can significantly influence the purchase behavior of air purifiers [76]. Whether people perceive air pollution as serious or not will affect their purchase behavior of related products [77]; non-smokers may focus on physical health [78] and may be more willing to choose indoor air purifiers [79]. Table 4 reports the results of the heterogeneity analysis.

According to Table 4, there are differences in the effect of indoor air purification in the results estimated by the generalized ordered logit model. The coefficients of indoor air purification with significant statistical significance appear in the comparative estimates of the “Good” group and the “Bad or Fair” group [rows (ii)]. These results show that, after the individual characteristics are grouped, the promotion effect of indoor air purification on self-rated health levels has little impact on movements between “Bad” and “Fair”, and is mainly reflected in the improvement to the “Good” level. This point will no longer be emphasized in the detailed analysis of results in Table 4.

As shown in row (1) of Table 4, indoor air purification has a significant positive impact on the self-rated health of rural residents, but the effect on urban residents is not statistically significant. When the quantity of indoor air purification increases by one unit, the odds ratio of rural residents’ health level improving to “Good” becomes 1.517[=exp (0.417)] times that of the original level. We have found similar results for rural populations in other studies. Sereenonchai et al. [80] studied willingness to pay (WTP) for self-protection and haze management. They found that WTP for an air purifier was the highest in rural plain areas, followed by the urban area. Furthermore, the education factors may explain the willingness to pay for air purifiers in rural areas [81]. With the development of the economy and the improvement in people’s living standards in China, rural areas have gradually understood and been able to use air purifiers. In addition, rural housing uses more highly polluting fuels, such as coal, so rural people are more willing to use air purifiers.

As shown in row (2) of Table 4, indoor air purification has a significantly positive effect on self-rated health in the high-education group, but not in the low-education group. This result suggests that increasing the use of indoor air purifiers is less important for improving the self-rated health of low-education populations. A possible explanation is that people with high education levels generally have a higher level of awareness of indoor air purification and easily gain knowledge about indoor air purifiers, so individuals in this group have a greater willingness to use air purification and believe that it can effectively improve their own health.

As shown in row (3) of Table 4, the effect of indoor air purification on the self-rated health of the non-working group is significantly positive at the 5% level, but not significant for the working group. This result shows that people without jobs pay more attention to the air quality in their houses because they are at home for a long time, and believe that air purifiers have a positive impact on their own health. As shown in row (4) of Table 4, the effect of indoor air purification on the self-rated health of non-smokers is significantly positive at the 5% level, but the effect on smokers is not significant, and the coefficient is negative. This result indicates that non-smokers pay more attention to indoor air quality and their own lung health, whereas smokers tend to care less about these and believe that even the use of air purifiers will not improve their health.

### 4.3. Robustness Check

#### 4.3.1. Changing the Methods of the Estimation Model

Considering the inaccurate estimation results due to the selection bias of the link function, we used the ordered probit model and the generalized ordered probit model to re-examine the effect of indoor air purification on self-rated health. The results of columns (1) and (2) of Table A1 in the Appendix A show that, in the ordered probit model [column (1)], the coefficient of indoor air purification is significantly positive at the 10% level, which has a positive impact on self-rated health. In the generalized ordered probit model [column (2)], the coefficient of indoor air purification is still different between the two groups, and the promotion effect of indoor air purification on the self-assessed health level is still mainly reflected in the improvement to the “Good” level. There is not much movement between “Bad” and “Fair” levels. This result is consistent with the conclusions of this study.

#### 4.3.2. Changing the Sample

Considering that some older people have chronic diseases and lower self-rated health levels due to the decline in physical function, the use of indoor air purifiers is likely to have little effect on the health improvement of these groups. Therefore, we further narrowed the sample range and selected samples younger than 60 years old. The results in column (3) of Table A1 in the Appendix A show that indoor air purification has a positive effect on self-rated health, and that this effect is more statistically significant (5% significance level). In the multivariate ordinal logit model, the coefficient of indoor air purification is also different between the two groups, and the promotion effect of indoor air purification on the self-assessed health level is still mainly reflected in the improvement to the “Good” level.

#### 4.3.3. Replacing the Dependent Variable

This study mainly analyzed the relationship between indoor air purification and self-rated health. However, considering the limitations of self-assessment of health, we define an indicator “Health_object” to objectively evaluate the health level [from the “Health Services and Disease History” part of the CHNS questionnaire, specifically the question “During the past 4 weeks, have you been sick or injured? Have you suffered from a chronic or acute disease?” (options: “0 = No”; “1 = Yes”; “9 = Unknown”)]. We removed those who answered “Unknown” from the sample. We also exchanged the signs of 0 and 1 in order to measure the objective health level. Since the objective health level index is a binary variable, we used the basic logit model. The results in the last column of Table A1 in the Appendix A show that, although the results of the coefficients are not significant, the conclusion that indoor air purification has a positive effect on the objective health level is still confirmed. The reason why it is not significant may be that the evaluation index of the objective health level is only measured by whether the patient is diagnosed with a disease, rather than the measurement of a comprehensive physical examination, resulting in a relatively single evaluation standard. However, self-rated health is a comprehensive evaluation of people’s own physical condition. Therefore, the validity and robustness of self-assessed health cannot be denied.

## 5. Conclusions

The impact of indoor air purification on residents’ health has been of interest to scholars, but empirical evidence from large samples is lacking. Based on 2015 CHNS data and a generalized ordinal logit model, we investigated the causal relationship between indoor air purification in housing and residents’ self-rated health. The results show that indoor air purification has a positive effect on residents’ self-rated health, but this effect is limited to improving the probability of residents’ health level to “Good”, and there is no significant movement between the two levels of “Bad” and “Fair”. If the number of air purifiers increases by one unit, the odds ratio of self-rating becoming “Good” is 1.236 times higher than before. Our research also found that solid fuels used in cooking, as a significant source of indoor air pollutants, significantly reduces residents’ self-rated health.

In addition, we also found that heterogeneity between different characteristic groups also affects the relationship between indoor air purification and residents’ self-rated health. The results of the heterogeneity analysis showed that, due to differences in housing conditions, indoor air purification has a significant positive impact on the self-rated health of rural residents, but the effect on urban residents is not statistically significant. The use of air purifiers has a greater impact on the health of low-education individuals. Due to going out for work, the impact of indoor air purification on the health of non-working residents is greater than that for working residents. Furthermore, non-smokers pay more attention to indoor air purification and personal health than smokers.

From a policy perspective, this study demonstrates the need for greater popularization and subsidy incentives for indoor air purification in order to control the health risks caused by indoor pollutants. Furthermore, considering the heterogeneity between groups with different characteristics, the government and other departments can formulate targeted policies to guide them to pay attention to indoor air purification, such as providing a cleaner working environment for urban residents who are busy with work. Furthermore, rural residents should be given more subsidies for indoor air purification, as the use of indoor air purifiers is more beneficial.

This study has some limitations. As the data from the China Health and Nutrition Survey are only updated to 2015, we are not able to obtain the latest data and further discuss the possible effect of indoor air purification on the infectivity of COVID-19. Moreover, due to limited data, we did not examine the impact of more sources of indoor pollutants and particulate matter, and/or gaseous pollutant concentrations on residents’ health; thus, this study cannot provide a better examination of the impacts of this potential mechanism. These limitations can be used as the main direction for future research.

## Figures and Tables

**Table 1:** Descriptive statistics of the variables.

Variables	Definition	*N*	Mean	SD	Min	Max
Health_self	The residents’ self-rated health status: 1 = Bad; 2 = Fair; 3 = Good	9612	2.506	0.601	1	3
Air_purifier	Number of air purifiers owned by the household	9612	0.038	0.192	0	1
Indoor living conditions						
HHarea	Housing size (area)	9612	141.550	115.621	15	960
Fuel	1 = Household uses solid fuels; 0 = Other fuels	9612	0.114	0.318	0	1
Lighting	Type of lighting generally used by household	9612	0.997	0.057	0	1
Demographics						
Age	Age of the person (years)	9612	49.697	15.009	15	94
Gender	1 = Male; 0 = Female	9612	0.509	0.500	0	1
Married	1 = Yes; 0 = No	9612	0.900	0.300	0	1
Size	Number of household members	9612	4.434	2.264	1	17
Socioeconomic status						
Edprim	1 = Lower middle school degree; 0 = Other	9612	0.558	0.497	0	1
Edmid	1 = Upper middle school degree or vocational degree; 0 = Other	9612	0.268	0.443	0	1
Edhigh	1 = University degree or higher; 0 = Other	9612	0.171	0.377	0	1
Work	1 = Yes; 0 = No	9612	0.508	0.500	0	1
Income (yuan)	Per capita annual household income	9612	18,108.470	23,207.910	0	666,667
Individual health beliefs						
Smoke	1 = Yes; 0 = No	9612	0.287	0.529	0	9
Drink_f	5 = almost every day; 4 = 3–4 times a week; 3 = once or twice a week; 2 = once or twice a month; 1 = no more than once a month; 0 = Never	9612	0.920	1.623	0	5
Activities	1 = Participation in physical activities; 0 = No	9612	0.160	0.367	0	1
PHS	1 = Utilization of preventive health care (PHS); 0 = No	9612	0.041	0.198	0	1
MI	1 = Participation in medical insurance (MI); 0 = No	9612	0.973	0.163	0	1
Community-enabling factors						
City	1 = Household in an urban area; 0 = Household in a rural area	9612	0.422	0.494	0	1
URBAN	Urbanization index	9612	75.544	16.746	31.458	104.400
SANI	Sanitation score	9612	7.552	2.236	0.3	10.000

**Table 2:** Regression results of the effect of indoor air purification on residents’ self-rated health based on the ordinal logit model.

Variables	Health_self			
	(1)	(2)	(3)	(4)
Air_purifier	0.507 ***	0.476 ***	0.237 *	0.211 *
	(0.112)	(0.113)	(0.117)	(0.117)
HHarea		0.001 ***	0.001 ***	0.001 ***
		(0.0002)	(0.0002)	(0.0002)
Fuel		−0.368 ***	−0.200 ***	−0.128 *
		(0.062)	(0.064)	(0.067)
Lighting		0.392	0.342	0.310
		(0.351)	(0.354)	(0.353)
Gender			−0.024	−0.019
			(0.048)	(0.049)
Age			−0.017 ***	−0.018 ***
			(0.002)	(0.002)
Married			0.172 **	0.183 ***
			(0.068)	(0.068)
Size			−0.044 ***	−0.041 ***
			(0.010)	(0.010)
Edprim			-0.0003	−0.001
			(0.051)	(0.051)
Edmid			0.214 ***	0.186 ***
			(0.064)	(0.064)
Edhigh			0.427 ***	0.381 ***
			(0.071)	(0.072)
Work			0.149 ***	0.159 ***
			(0.046)	(0.046)
Income			1.93 × 10^−6^ **	1.59 × 10^−6^
			(1.02 × 10^−6^)	(1.05 × 10^−6^)
Smoke			−0.036	−0.025
			(0.045)	(0.046)
Drink_f			0.0012	0.0002
			(0.013)	(0.013)
Activities			0.298 ***	0.287 ***
			(0.059)	(0.059)
PHS			−0.308 ***	−0.326 ***
			(0.104)	(0.104)
MI			−0.260 **	−0.244 *
			(0.131)	(0.131)
City				0.008
				(0.050)
URBAN				0.0001
				(0.002)
SANITATION				0.051 ***
				(0.013)
/cut1	−2.819 ***	−2.400 ***	−3.376 ***	−3.008 ***
	(0.045)	(0.354)	(0.398)	(0.410)
/cut2	−0.226 ***	0.201	−0.711 *	−0.337
	(0.021)	(0.352)	(0.395)	(0.408)
Observations	9612	9612	9612	9612
Log likelihood	−8280.79	−8157.79	−7955.16	−7940.50
Pseudo R2	0.001	0.004	0.030	0.031

Note: *, **, and *** refer to the 10%, 5%, and 1% significance levels, respectively.

**Table 3:** Regression results of the effect of indoor air purification on residents’ self-rated health based on the generalized ordinal logit model.

Variables	Health_self							
	(1)		(2)		(3)		(4)	
	(i)	(ii)	(i)	(ii)	(i)	(ii)	(i)	(ii)
Air_purifier	0.575 **	0.503 ***	0.546 *	0.472 ***	0.306	0.228 *	0.311	0.212 *
	(0.297)	(0.113)	(0.298)	(0.114)	(0.305)	(0.118)	(0.306)	(0.118)
HHarea			0.001 *	0.001 ***	0.001 **	0.001 ***	0.001**	0.001 ***
			(0.0004)	(0.0002)	(0.0005)	(0.0002)	(0.0005)	(0.0002)
Fuel			−0.319 **	−0.375 ***	−0.100	−0.213 ***	−0.032	−0.143 **
			(0.126)	(0.065)	(0.130)	(0.067)	(0.136)	(0.069)
Lighting			0.540	0.360	0.412	0.311	0.396	0.279
			(0.610)	(0.363)	(0.622)	(0.367)	(0.621)	(0.367)
Gender					0.090	−0.036	0.093	−0.032
					(0.105)	(0.050)	(0.106)	(0.050)
Age					−0.022 ***	−0.016 ***	−0.023 ***	−0.017 ***
					(0.004)	(0.002)	(0.004)	(0.002)
Married					0.153	0.173 **	0.163	0.183 ***
					(0.139)	(0.070)	(0.139)	(0.070)
Size					−0.035 *	−0.044 ***	−0.032	−0.040 ***
					(0.020)	(0.010)	(0.021)	(0.010)
Edprim					0.161	−0.027	0.157	−0.028
					(0.105)	(0.052)	(0.105)	(0.052)
Edmid					0.332 **	0.192 ***	0.304 **	0.165 **
					(0.143)	(0.065)	(0.143)	(0.065)
Edhigh					0.408 **	0.421 ***	0.371 **	0.376 ***
					(0.175)	(0.072)	(0.179)	(0.073)
Work					0.446 ***	0.117 **	0.452 ***	0.127 ***
					(0.105)	(0.047)	(0.106)	(0.047)
Income					1.43 × 10^−5^ ***	1.35 × 10^−6^	1.53 × 10^−5^ ***	1.02 × 10^−6^
					(3.67 × 10^−6^)	(1.01 × 10^−6^)	(4.02 × 10^−6^)	(1.04 × 10^−6^)
Smoke					−0.072	−0.030	−0.057	−0.020
					(0.093)	(0.046)	(0.094)	(0.047)
Drink_f					0.030	-0.002	0.029	-0.003
					(0.029)	(0.013)	(0.029)	(0.013)
Activities					0.051	0.316***	0.047	0.306 ***
					(0.133)	(0.060)	(0.133)	(0.060)
PHS					−0.566 ***	−0.258 **	−0.586 ***	−0.275 ***
					(0.181)	(0.106)	(0.181)	(0.107)
MI					−0.225	−0.259 *	−0.219	−0.244 *
					(0.314)	(0.133)	(0.315)	(0.133)
City							−0.105	0.009
							(0.114)	(0.051)
URBAN							−0.0009	0.0001
							(0.004)	(0.002)
SANITATION							0.067 **	0.050 ***
							(0.029)	(0.013)
Constant	2.818 ***	0.227 ***	2.207 ***	−0.166	3.085 ***	0.753 *	2.700 ***	0.391
	(0.045)	(0.021)	(0.611)	(0.364)	(0.746)	(0.409)	(0.771)	(0.421)
Observations	9612	9612	9612	9612	9612	9612	9612	9612
Log likelihood	–8180.75		–8157.38		–7928.59		–7913.57	
Pseudo R2	0.001		0.004		0.032		0.034	

Note: *, **, and *** refer to the 10%, 5%, and 1% significance levels, respectively.

**Table 4:** Regression results of heterogeneity analysis of the effect of indoor air purification on residents’ self-rated health based on the generalized ordinal logit model.

Group	Model		Coefficient	SD	Covariates	Observations	Log Likelihood	Pseudo R2
(1)	Urban	(i)	0.345	0.357	Yes	4059	–3279.09	0.030
		(ii)	0.147	0.137	Yes	4059		
	Rural	(i)	0.171	0.604	Yes	5553	–4598.70	0.043
		(ii)	0.417 *	0.239	Yes	5553		
(2)	High education	(i)	0.244	0.418	Yes	3279	–2454.42	0.032
		(ii)	0.277 *	0.152	Yes	3279		
	Low education	(i)	0.448	0.468	Yes	6333	–5443.18	0.026
		(ii)	0.183	0.189	Yes	6333		
(3)	Work	(i)	0.165	0.484	Yes	4887	–3748.94	0.032
		(ii)	0.083	0.158	Yes	4887		
	Nowork	(i)	0.460	0.401	Yes	4725	–4131.19	0.031
		(ii)	0.373 **	0.179	Yes	4725		
(4)	Smoke	(i)	−0.326	0.503	Yes	2665	–2202.03	0.039
		(ii)	−0.064	0.248	Yes	2665		
	Nosmoke	(i)	0.613	0.394	Yes	6937	–5680.13	0.035
		(ii)	0.218 **	0.136	Yes	6937		

Note: *, and ** refer to the 10% and 5% significance levels, respectively.

## Data Availability

The datasets used and analyzed during the current study are available from the corresponding author on request.

## Appendix A

**Table A1:** Robustness check of the effect of indoor air purification on residents’ self-rated health.

Variables	Health_self						Health_object
	(1)	(2)		(3)	(4)		(5)
		(i)	(ii)		(i)	(ii)	
Air_purifier	0.129 *	0.126	0.125 *	0.290 **	0.355	0.285 **	0.080
	(0.069)	(0.140)	(0.072)	(0.141)	(0.402)	(0.141)	(0.171)
HHarea	0.0005 ***	0.0005 **	0.0005 ***	0.0007 ***	0.0010 *	0.0006 ***	0.0005
	(0.0001)	(0.0002)	(0.0001)	(0.0002)	(0.0006)	(0.0002)	(0.0003)
Fuel	−0.073 *	−0.022	−0.092 **	−0.201 **	−0.097	−0.214 **	0.109
	(0.040)	(0.067)	(0.043)	(0.081)	(0.181)	(0.084)	(0.112)
Lighting	0.201	0.236	0.181	0.337	0.843	0.227	−0.065
	(0.208)	(0.328)	(0.228)	(0.403)	(0.625)	(0.407)	(0.622)
Gender	−0.007	0.043	−0.020	−0.030	−0.062	−0.037	0.116
	(0.029)	(0.052)	(0.031)	(0.059)	(0.141)	(0.060)	(0.075)
Age	−0.011 ***	−0.011 ***	−0.011 ***	−0.022 ***	−0.033 ***	−0.021 ***	−0.030 ***
	(0.001)	(0.002)	(0.001)	(0.002)	(0.006)	(0.002)	(0.003)
Married	0.109 ***	0.091	0.116 ***	0.233 ***	0.395 **	0.220 ***	−0.051
	(0.041)	(0.068)	(0.044)	(0.083)	(0.183)	(0.085)	(0.109)
Size	−0.024 ***	−0.017 *	−0.025 ***	−0.023 *	−0.002	−0.024 *	0.005
	(0.006)	(0.010)	(0.006)	(0.013)	(0.030)	(0.013)	(0.016)
Edprim	0.005	0.077	−0.018	0.042	0.197	0.027	0.052
	(0.030)	(0.050)	(0.033)	(0.064)	(0.145)	(0.065)	(0.081)
Edmid	0.117 ***	0.152 **	0.104 **	0.207 ***	0.328 *	0.193 **	0.060
	(0.038)	(0.067)	(0.040)	(0.078)	(0.183)	(0.079)	(0.100)
Edhigh	0.225 ***	0.159 **	0.229 ***	0.399 ***	0.236	0.401 ***	−0.033
	(0.043)	(0.079)	(0.045)	(0.085)	(0.228)	(0.086)	(0.109)
Work	0.105 ***	0.214 ***	0.079 ***	0.222 ***	0.483 ***	0.195 ***	0.157 **
	(0.027)	(0.049)	(0.029)	(0.054)	(0.126)	(0.055)	(0.075)
Income	1.11 × 10^−6^ *	7.23 ×10^−6^ ***	5.83 × 10^−7^	1.89 × 10^−6^	2.44 × 10^−5^ ***	1.22 × 10^−6^	4.03 × 10^−6^
	(5.79 × 10^−7^)	(1.98 × 10^−6^)	(6.98 × 10^−7^)	(1.30 × 10^−6^)	(5.75 × 10^−6^)	(1.28 × 10^−6^)	(2.52 × 10^−6^)
Smoke	−0.017	−0.029	−0.014	−0.035	−0.080	−0.031	−0.119 **
	(0.026)	(0.048)	(0.030)	(0.054)	(0.123)	(0.055)	(0.058)
Drink_f	0.001	0.014	-0.002	-0.005	0.006	-0.005	−0.012
	(0.008)	(0.013)	(0.008)	(0.015)	(0.036)	(0.015)	(0.020)
Activities	0.160 ***	0.021	0.189 ***	0.283 ***	−0.102	0.307 ***	−0.019
	(0.035)	(0.063)	(0.037)	(0.071)	(0.165)	(0.071)	(0.090)
PHS	−0.203 ***	−0.295 ***	−0.171 **	−0.196	−0.677 **	−0.136	−1.278 ***
	(0.061)	(0.094)	(0.067)	(0.144)	(0.269)	(0.145)	(0.117)
MI	−0.134 *	−0.083	−0.146 *	−0.299 **	−0.153	−0.305 **	0.040
	(0.078)	(0.145)	(0.082)	(0.150)	(0.371)	(0.151)	(0.209)
City	0.001	−0.055	0.004	−0.072	−0.398 ***	−0.057	−0.446 ***
	(0.029)	(0.056)	(0.032)	(0.059)	(0.148)	(0.060)	(0.082)
URBAN	3.44 × 10^−5^	−0.0003	5.43 × 10^−5^	−0.0001	0.0023	−0.0005	0.0065 **
	(0.001)	(0.002)	(0.001)	(0.002)	(0.005)	(0.002)	(0.003)
SANITATION	0.031 ***	0.032 **	0.031 ***	0.032 **	0.050	0.032 ^**^	−0.037 *
	(0.008)	(0.014)	(0.008)	(0.015)	(0.037)	(0.016)	(0.021)
/cut1	−1.675 ***			−3.315 ***			
	(0.240)			(0.476)			
/cut2	−0.179			−0.543			
	(0.239)			(0.473)			
Constant		1.454 ***	0.237		2.167 **	0.710	3.423 ***
		(0.400)	(0.262)		(0.868)	(0.478)	0.720
Observations	9612	9622	9622	6923	6923	6923	9612
Log likelihood	−7935.31	–7913.31		−5500.91	–5478.02		−3297.69
Pseudo R2	0.031			0.025	0.030		0.061

Note: *, **, and *** refer to the 10%, 5%, and 1% significance levels, respectively.
